# Response to transarterial chemoembolization may serve as selection criteria for hepatocellular carcinoma liver transplantation

**DOI:** 10.18632/oncotarget.20511

**Published:** 2017-08-24

**Authors:** Jianyong Lei, Jinjing Zhong, Yefang Luo, Lunan Yan, Jinqiang Zhu, Wentao Wang, Bo Li, Tianfu Wen, Jiaying Yang, Liver Surgery Group

**Affiliations:** ^1^ Department of Liver Surgery, West China Hospital of Sichuan University, Chengdu, China; ^2^ Thyroid and Parathyroid Surgery Center, West China Hospital of Sichuan University, Chengdu, China; ^3^ Transplantation Center,West China Hospital of Sichuan University, Chengdu, China; ^4^ Department of Pathology, West China Hospital of Sichuan University, Chengdu, China; ^5^ West China School of Medicine, Sichuan University, Chengdu, China; ^6^ Present address: Liver Surgery, West China Hospital of Sichuan University, Chengdu, China

**Keywords:** hepatocellular carcinoma, liver transplantation, transarterial chemoembolization, selection

## Abstract

**Aims:**

This study sought to extend the inclusion criteria for hepatocellular carcinoma (HCC) liver transplantation (LT), particularly addressing the safety and effectiveness of pre-LT transarterial chemoembolization (TACE).

**Materials and Methods:**

Our study included 115 patients with HCC who underwent LT after TACE. The response measured after each TACE session was based on the mRECIST criteria: complete response (CR), partial response (PR), stable disease (SD) or progressive disease (PD). We defined CR and PR patients as responders (64 cases) and SD and PD patients as non-responders (51 cases).

**Results:**

The majority of responders could be identified after the first or second TACE sessions (57 cases, 89.1%). Overall survival rates at 1, 3 and 5 years were 95.3%, 89.1% and 75.0%, respectively, in the responder group, and these rates were much higher than those in the non-responder group (86.3%, 66.7% and 54.9%, P=0.016). In addition, the tumor-free survival rate in the responder group was also higher than in the non-responder group (P=0.009). In the responder group, a statistically improved long-term outcome was observed in patients whose HCC did not satisfy the Milan criteria (P<0.05). Univariate and multivariate Cox analyses showed that achieving CR or PR was the best predictor of survival and tumor-free survival following TACE.

**Conclusion:**

The response to TACE, particularly following the first two sessions, primarily and robustly predicted overall and tumor-free survival in HCC patients, particularly those whose HCC did not satisfy the Milan criteria.

## INTRODUCTION

Hepatocellular carcinoma (HCC) is the third-leading cause of cancer-related deaths worldwide, with approximately 750,000 new cases of liver cancer [1] and 1 million deaths [2] reported each year. Between 60 and 90% of HCC patients are diagnosed in association with liver cirrhosis [3], and liver transplantation (LT) is one of the most effective treatments for HCC. Although this approach eliminates both the cancer and premalignant cirrhotic liver tissue, it remains controversial whether LT should be the ideal treatment option for different stages of HCC because recurrence is a major consideration and the most common cause of mortality in transplanted patients [3]. Initially, the results of LT in HCC patients were disappointing, with high recurrence rates and dismal patient survival due to the advanced state of disease at the time of transplantation. Indeed, the United Network for Organ Sharing (UNOS) once considered HCC a contraindication for LT [4]. However, in 1996, Mazzaderro et al.[5] demonstrated that patients with cirrhosis and a single HCC of up to 5cm or up to three tumors of which none were larger than 3cm and with no evidence of macrovascular invasion or extrahepatic spread showed a 4-year post-transplant survival that was similar to patients with non-malignant disease; in addition, the 5-year survival rate was 61.1% in comparison to the previously observed rate of 25.3% in 1987. Subsequently, these criteria have been referred to as the Milan criteria. The guidelines of the European Association for the Study of the Liver (EASL), the American Association for the Study of the Liver Disease (AASLD) and AASLD recommend that LT for HCC should be performed in patients meeting these criteria [3]. Although many other criteria have attempted to include more HCCs that do not satisfy the Milan criteria, there is no consolidated upper limit. The selection items for HCC LT primarily include data on tumor histology, including tumor number, diameter, volume, histological level and microvascular invasion [5-8] (e.g., the Milan criteria, the UCSF criteria and the Hangzhou criteria), serum biomarkers such as alpha-fetoprotein (AFP) and micro-RNAs [7, 9] (e.g., the Hangzhou criteria) and the response to transarterial chemoembolization(TACE)[10]. However, none of these biological features are routinely available or definitively characterized prior to transplantation [11]. Moreover, the majority of published inclusion criteria are based on analyses of explanted livers, which provide information that is not available prior to surgery when the decision for LT is made for an HCC patient, as well as predictions of the size and number of nodules, which are based upon imaging and may differ from the actual size and number in 30-60% of samples [10, 12, 13]. Due to its relative safety, efficacy and reproducibility, TACE has been introduced and performed extensively not only for palliation but also as a pre-operative adjuvant [14]. In our previous study, we have proved response to TACE may serve as a selection criterion for resection of Barcelona Clinic Liver Cancer (BCLC) stage B HCC. Thus, the response to pre-LT TACE may represent a feasible inclusion criterion for HCC LT. However, the choice of pre-LT therapy remains difficult, and the effectiveness over the long term remains controversial [15]. We hypothesized that changes in tumor features resulting from pre-transplant adjuvant TACE may constitute a superior criterion for predicting tumor recurrence; therefore, this study was conducted to evaluate the efficacy and safety of pre-LT adjuvant TACE when used as an inclusion criterion.

## RESULTS

### TACE toxicity

Data on the toxicity of TACE were graded based on World Health Organization criteria. In the majority of the TACE group, treatment was well tolerated, and the most significant toxicities associated with TACE were transient hepatic toxicity or hepatic function impairment in 87 cases (75.7%), with minor toxicity (grade 1) observed in the majority of these cases (82, 94.3%); pain in the upper quadrant (58, 50.4%); nausea/emesis (51, 44.3%) and fever (41, 35.7%). A grade 3 adverse reaction developed in 8 of 115 patients (7.0%), and no grade 4 adverse reactions occurred, as shown in Table 1. Only a small portion of patients (28, 24.3%) suffered no adverse events. Meanwhile, we did find any associations beween TACE toxicity and post-LT survival.

**Table 1:** Adverse events following TACE

Adverse reactions (%)	Grade 1	Grade 2	Grade 3	Grade 4
Nausea/emesis	48 (41.7%)	3 (2.6%)	0	0
Fever	35 (30.4%)	5 (4.3%)	1(0.9%)	0
Pain in the upper quadrant	51 (44.3%)	6 (5.2%)	1(0.9%)	0
Ischemic liver function impaired	82 (71.3%)	3(2.6%)	2(1.7%)	0
Femoral artery pseudoaneurysm	0	0	1(0.9%)	0
Spontaneous bacterial peritonitis	0	0	1 (0.9%)	0
Allergy	0	2(1.7%)	1(0.9%)	0
Sepsis	0	0	1(0.9%)	0

Toxicity was graded based on World Health Organization criteria.

### Response to TACE

**Figure 1:**
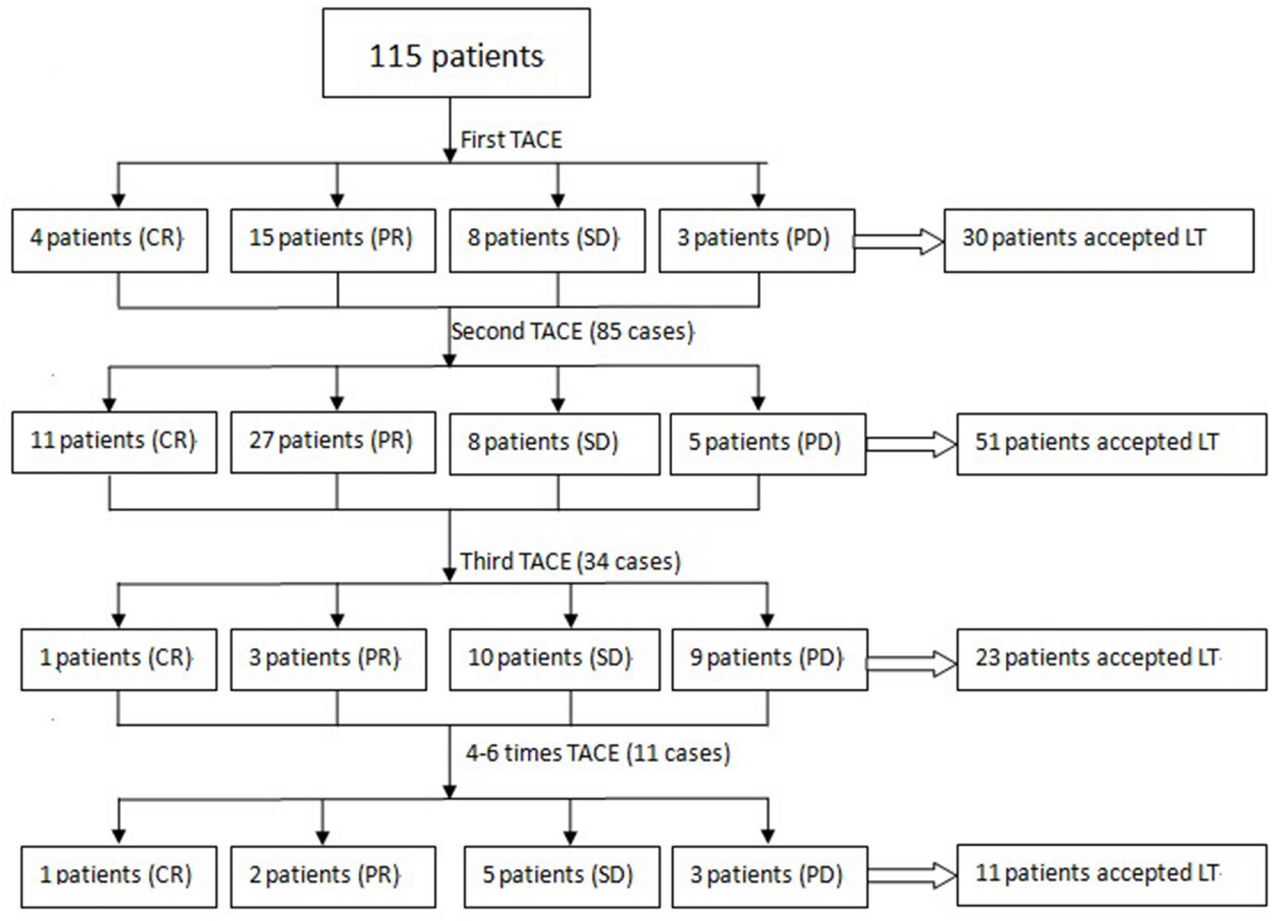
The majority of patients received TACE twice (51 patients), although TACE was administered once in 30 patients, three times in 23 patients, and more than three times in 11 patients A response was recorded in 19 patients after one session of TACE, in 38 patients after a second session, in 4 patients after a third session, and in only 3 patients when over four sessions were used.

LT was performed 33-351 days after TACE (135.2± 60.2 days). The number of TACE sessions in the 115 patients ranged from 1 to 6 (2.0±0.9), with the majority of patients treated twice (51 patients), 30 patients treated once, 23 patients treated three times, and 11 patients undergoing TACE more than three times. Based on the final radiological assessment prior to LT, 17 patients (14.8%) showed no sign of variable tumor (CR), 47 patients (40.9%) showed more than a 30%reduction in viable tumor (PR), 31 patients (27.0%) were stable (SD), and 20 patients (17.4%) showed PD despite TACE. The 115 patients were then assigned to the ‘responder’(CR or PR, 64 patients) or ‘non-responder’ (SD or PD, 51 patients) group. As shown in Figure 1, after initial TACE, even with 8 SD patients and 3 PD patients, the HCCs all satisfied the Hangzhou criteria, and LT proceeded due to the availability of liver grafts. Thirteen patients who were non-responders also accepted LT after the second TACE, 19 patients after the third TACE, and 8 patients after receiving TACE 4 to 6 times. The majority of HCC responders (CR or PR) could be identified after the first or second TACE sessions. The responders included 19 of the 115 patients (16.5%) after the first TACE, 38 of 85patients (44.7%) after the second TACE, 4 of 34 patients (11.8%) after the third TACE, and 3of 11 patients (27.3%) who received TACE more than three times (as shown in Figure 1).

### Baseline characteristics of the recipients and donors

The baseline demographics of the two groups (responders and non-responders) are shown in Table 2. The majority of the recipients were male (103 cases, 89.6%), and HBV was the most common cause of HCC in our study (106 cases, 92.1%). No significant difference was observed between the two groups with respect to baseline characteristics of the recipients. Although 10 patients in the responder group and 6 patients in the non-responder group showed Child-Pugh C liver function, liver function in the majority of the recipients was tolerant when the LT proceeded, and liver function in the two groups was comparable. There was no significant difference in recipient intraoperative characteristics or donor characteristics. The non-responder group underwent more sessions of TACE than the responder group (1.9±0.9 vs. 2.5±1.0, P=0.001).

**Table 2:** Baseline characteristics of the patients and donors

	Responder group	Non-responder group	P value
	64	51	
Age (years)	45.4±10.2	45.1±9.9	0.899
Gender (M/F)	55/9	48/3	0.156
Weight (Kg)	65.2±9.6	67.0±10.7	0.357
Height (cm)	167.8±6.6	165.8±4.3	0.541
BMI (kg/m^2^)	23.0±2.7	23.2±3.2	0.803
Blood type (A/B/AB/O)	21/13/20/10	19/16/11/5	0.210
Cirrhosis etiology (HBV/HCV/others)	59/1/4	47/1/3	0.885
MELD score	10.6±4.8	9.8±3.7	0.370
Child-Pugh score (A/B/C)	25/29/10	16/29/6	0.675
Liver graft (DDLT/LDLT)	55/9	48/3	0.156
Intraoperative blood loss (ml)	1721.1±799.7	1695.1±631.5	0.850
Operative time (hours)	9.0±2.1	8.8±2.6	0.555
Intraoperative infusion volume (ml)	7717.0±5326.5	8098.9±6053.3	0.720
Donor age (years)	32.1±9.3	32.3±3.0	0.905
Donor BMI (kg/m^2^)	21.8±2.1	22.7±3.0	0.164
Waiting days (from first TACE to LT)	140.9±54.1	128.1±67.0	0.284
TACE times (1/2/3/multiple)	19/38/4/3	11/13/19/8	0.001^*^

BMI: body mass index; HBV: hepatitis B virus; HCV: hepatitis C virus; MELD: Model for end-stage liver disease; DDLT: deceased-donor liver transplantation; LDLT: living-donor liver transplantation; TACE: transarterial chemoembolization; LT: liver transplantation

### Factors contributing to the TACE response

Although the baseline patient characteristics and liver function levels did not contribute to TACE responses, a comparison of responder and non-responder tumor characteristics was performed, as shown in Table 3. Based on the evaluation criteria, multiple tumor targets, an absence of obvious arterial-phase enhancement during preoperative imaging, microvascular invasion and a neutrophil-lymphocyte ratio (NLR)≥4 were found to contribute to non-responsiveness (greater target number in the imaging scan or explanted liver, less cases with arterial-phase enhancement during the imaging scan, more cases with microvascular invasion, and more cases showing NLR≥4; all P<0.05). Other factors, such as the largest and second-largest target diameter, total diameter of all targets, tumor within or outside the Milan or UCSF criteria and tumor differentiation, did not differ significantly between the two groups (Table 3). The targets in 10 patients were necrotic, and no tumor specimen was obtained; however, during pre-LT imaging evaluation, 17 patients were classified as CR. Thus, the HCCs in 7 patients were not necrotic, even in cases without a target based on imaging. At HCC diagnosis, the baseline AFP level was comparable between the two groups; however, after TACE, the responder group showed a greater decrease in AFP compared to the non-responder group. The average AFP level decreased from 1,588.2 ng/ml to 264.6 ng/ml in the responder group and from 899.1 ng/ml to 729.2 ng/ml in the non-responder group.

**Table 3:** Baseline characteristics of the tumors

	Responder group	Non-responder group	P value
	64	51	
Target number (solitary/2/3/multiple)	43/17/4/0	29/8/4/10	0.047^*^
Tumor diameter of the largest target (cm)	6.4±3.8	6.0±3.8	0.615
The diameter of the second-largest tumor target (cm)	2.7±1.6	3.3±2.4	0.404
Target number in explanted liver (solitary/2/3/multiple, new target)	38/20/5/1(8)	23/12/5/11(12)	0.016^*^
Total diameter of the tumor targets prior to TACE (cm)	6.8±4.2	6.8±3.9	0.989
Total diameter of the tumor targets prior to LT (cm)	5.2±3.4	5.1±3.6	0.885
Total diameter of the tumor targets in explanted liver (cm)	5.8±3.9	5.2±3.8	0.693
Obvious arterial-phase enhancement (yes/no)	40/24	21/30	0.023^*^
Milan criteria satisfied at TACE(yes/no)	34/30	23/28	0.394
UCSF criteria satisfied at TACE(yes/no)	42/22	32/19	0.750
AFP level (ng/ml) prior to TACE (A/B/C/D)	31/6/4/23	33/1/2/15	0.149
AFP level (ng/ml) when transplanted (A/B/C/D)	48/4/7/5	27/8/10/6	0.024^*^
NLR≥4 (yes/no)	19/45	27/24	0.012^*^
Microvascular invasion (yes/no)	14/50	26/25	0.001^*^
Tumor differentiation (well/moderate/poor/ON)	19/24/11/10	26/15/10/0	0.632

NLR: neutrophil-lymphocyte ratio; AFP level: A, 0-400ng/ml; B, 400-800ng/ml; C, 800-1,200 ng/ml; D, ≥1,210 ng/ml; TACE: transarterial chemoembolization; LT: liver transplantation; UCSF: University of California, San Francisco; AFP: alpha-fetoprotein; ON: overall necrosis

### Overall and tumor-free survival rate

**Figure 2:**
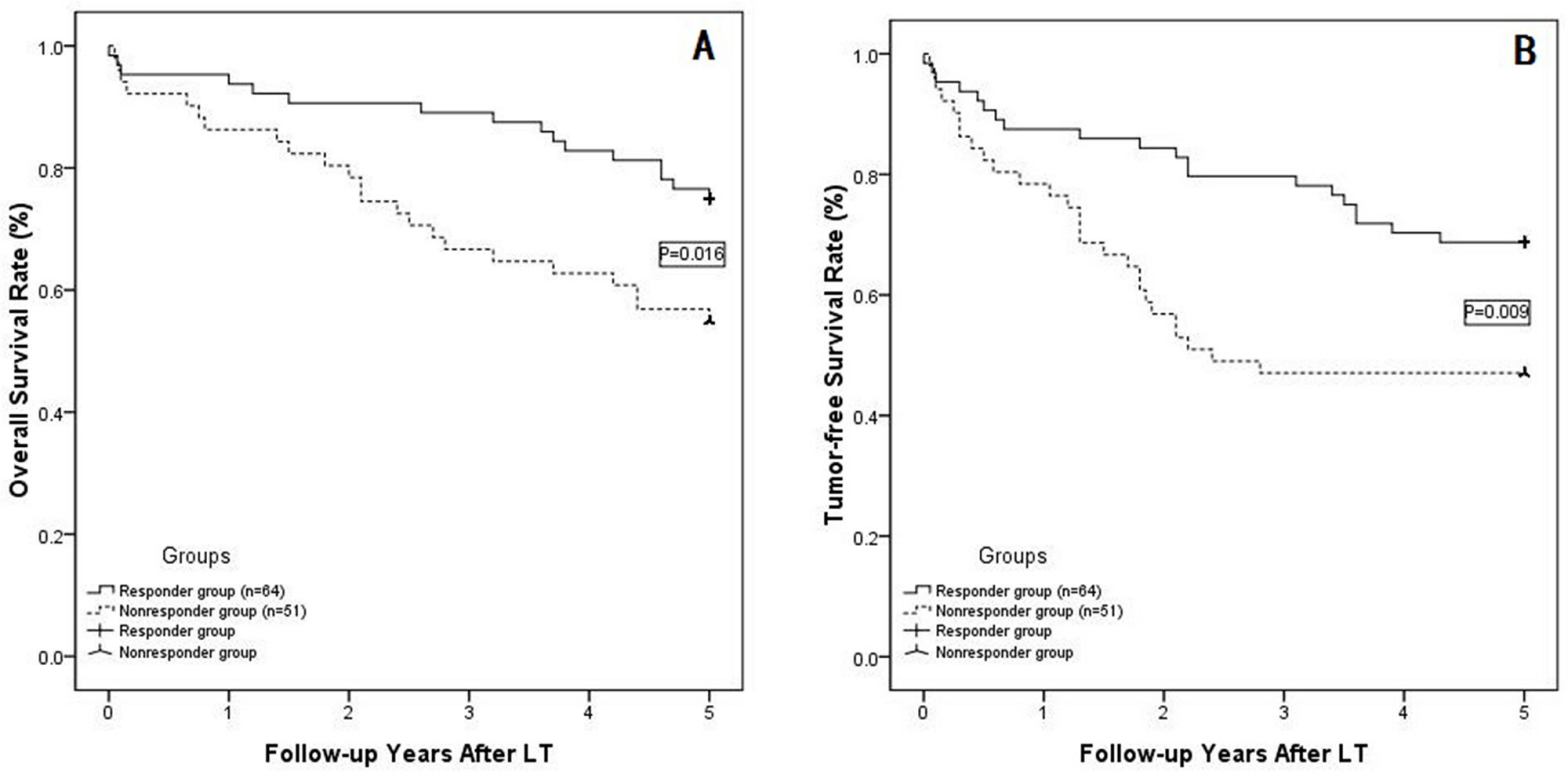
**(A)** The overall survival rates at 1, 3 and 5 years were 95. 3%, 89.1% and 75.0% in the responder group and 86.3%, 66.7% and 54.9% in the non-responder group (P=0.016), respectively; **(B)** The 1-, 3- and 5-year tumor-free survival rates in the responder group were 86.3%, 66.7% and 54.9%, respectively, and 78.4%, 47.1% and 47.1%, respectively, in the non-responder group (P=0.009).

During the last 5 years of follow up, 30 patients died, and the primary cause of death during follow-up was tumor recurrence (22 cases, 73.3%) followed by LT complications (7 cases, 23.3%) and a cardiovascular accident (1 case, 3.3%). Tumor recurrence or metastasis occurred in 40 patients, with the most-common site of tumor recurrence being the liver (27 cases, 67.5%), followed by liver recurrence and lung metastasis (4 cases, 10%), lung metastasis (3cases, 7.5%), intra-abdominal metastasis (3 cases, 7.5%), lung and bone metastasis (1 case, 2.5%), bone metastasis (1 case, 2.5%) and brain and lung metastasis (1 case, 2.5%). The cumulative overall survival rates at 1, 3 and 5 years were 95.3%, 89.1% and 75.0%, respectively, in the responder group, and these rates were much higher than those in the non-responder group (86.3%, 66.7%and 54.9%; P=0.016; as shown in Figure 2A). The 1-, 3- and 5-year tumor-free survival rates in the responder group (86.3%, 66.7% and 54.9%, respectively) were also higher than those in the non-responder group (78.4%, 47.1% and 47.1%, respectively; P=0.009;shown in Figure 2B).

### Subgroup analysis

**Figure 3:**
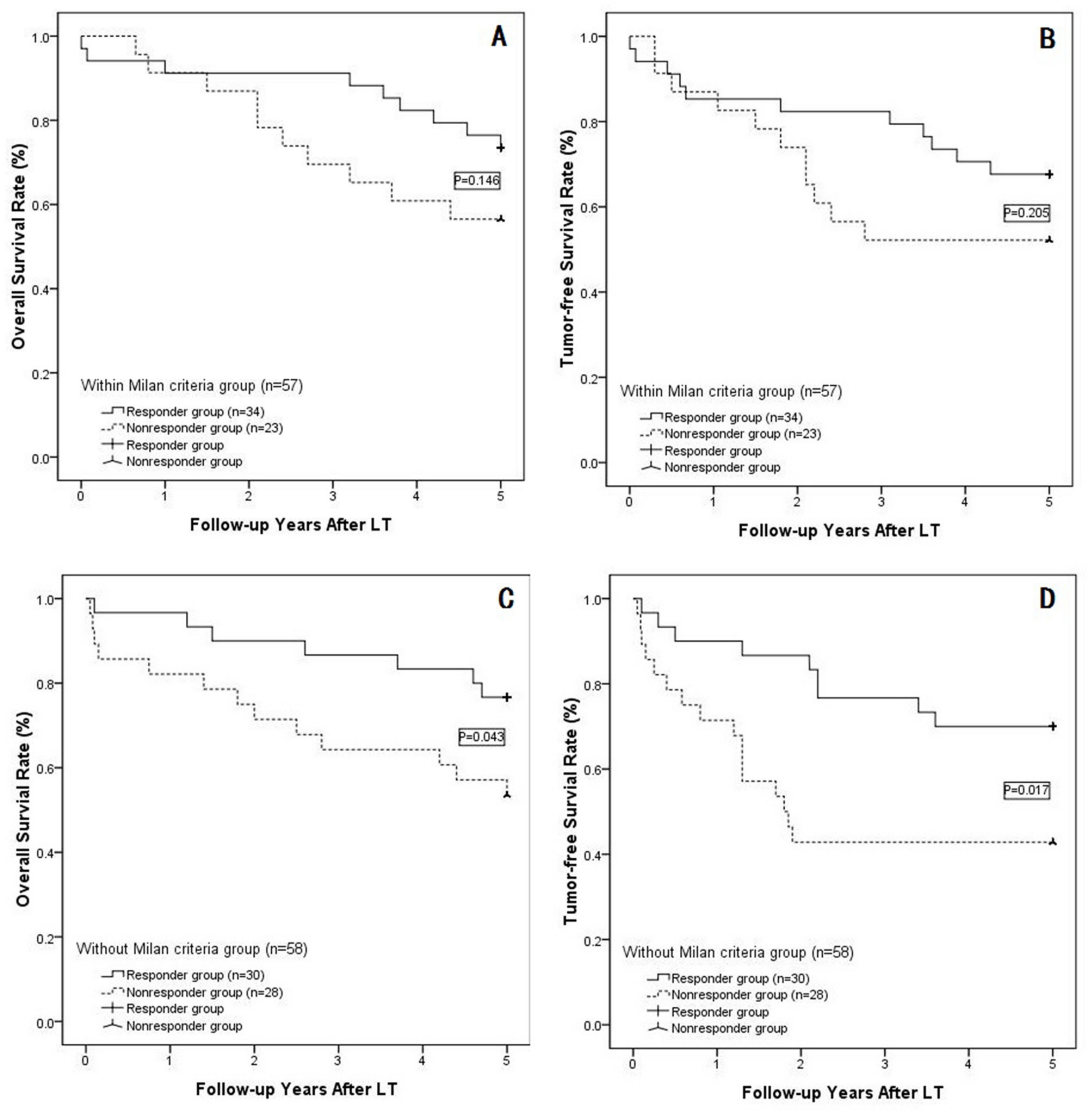
**(A)** In the subgroup of patients within the Milan criteria , the responders showed 1-, 3- and 5-year survival rates of 94. 1%, 91.2% and 76.5%, respectively, and non-responders showed cumulative 1-, 3- and 5-year overall survival rates of 91.3%, 70.0% 56.5% (P=0.146), respectively; **(B)** For those within the Milan criteria, the 1-, 3- and 5-year tumor-free survival rates were comparable between the responder and non-responder groups (P=0.205); **(C)** In the subgroup outside of the Milan criteria, the 1-, 3- and 5-year overall survival rates were 96.7%, 86.7% and 76.7%, respectively, in the responder group and 82.1%, 64.3% and 57.1%, respectively, in the non-responder group(P=0.043); **(D)** The 1-, 3- and 5-year tumor-free survival rates were 90.0%, 76.7% and 70.0%, respectively, in the responder group and 71.4%, 42.9% and 42.9%, respectively, in the non-responder group (P=0.017).

Based on histological examinations, we divided the 115 patients into two subgroups: those within the Milan criteria (57 cases) and those extending outside the Milan criteria (58 cases). Subgroup analyses were then performed within the responder group and non-responder group. Among patients within the Milan criteria, the responder group (34 cases) showed cumulative survival rates at 1, 3 and 5 years of 94.1%, 91.2% and 76.5%, respectively, and the non-responder group (23 cases) showed cumulative 1-, 3- and 5-year overall survival rates of 91.3%, 70.0% 56.5%, respectively; these two groups were statistically comparable (P=0.146; as shown in Figure 3A). The 1-, 3- and 5-year tumor-free survival rates were also comparable between responders and non-responders when evaluating patients within the Milan criteria (85.3%, 82.4% and 67.6%, respectively, in the responder group vs. 87.0%, 52.2% and 52.2%, respectively, in the non-responder group; P=0.205; as shown in Figure 3B). However, when the HCCs were outside of the Milan criteria (58 cases), responders showed much higher 1-, 3- and 5-year overall and tumor-free survival rates than the non-responder group (shown in Figure 3C and 3D); in particular, the 1-, 3- and 5-year overall survival rates were 96.7%, 86.7% and 76.7%, respectively, in the responder group and 82.1%, 64.3% and 57.1%, respectively, in the non-responder group (P=0.043). The 1-, 3- and 5-year tumor-free survival rateswere 90.0%, 76.7% and 70.0%, respectively, in the responder group and 71.4%, 42.9%% and 42.9%, respectively, in the non-responder group (P=0.017).

**Figure 4:**
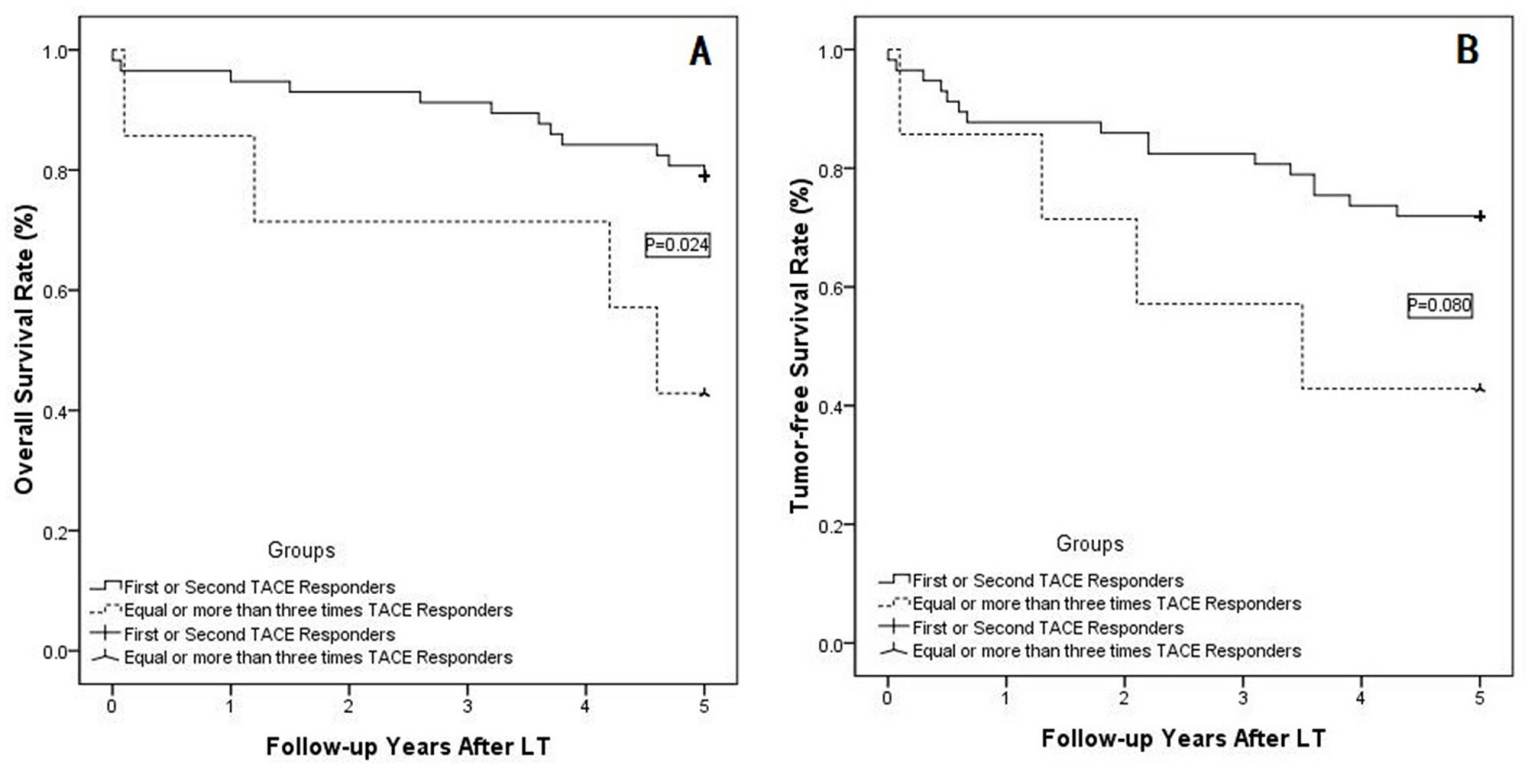
**(A)** In the responder subgroup, the patients who achieved a response after the first two TACE sessions showed much better 1-, 3- and 5-year overall survival rates than patients who achieved a response after 3 or more TACE sessions (P=0. 024); **(B)** The tumor-free survival rate was also better in responders to the first two TACE sessions compared with responders to 3 or more TACE sessions (P=0.080).

In the responder group, HCCs were classified as CR or PR (i.e., responders) in 19 patients after the initial TACE, in 38 patients after the second TACE, in 4 patients after the third TACE, and in 3 patients after 4-6 rounds of TACE (as shown in Figure 1). The overall survival rate and tumor-free survival rates were compared between individuals receiving 1 or 2 TACE treatments in the responder group and those receiving 3 or more TACE treatments in the responder group. The former group showed significantly improved 1-, 3- and 5-year overall survival rates than the latter group (P=0.024; as shown in Figure 4A). Moreover, the 1-, 3- and 5-year tumor-free survival rate was also better among responders after the first or second TACE treatment, although the P value was 0.080 (shown in Figure 4B).

### Univariate and multivariate analyses of overall and tumor-free survival

Additional survival analyses were performed and are shown in Tables 4 and 5; these analyses included factors associated with survival including recipient age, gender, body mass index (BMI), cause of liver disease, Child-Pugh score, albumin (ALB), creatinine, NLR, AFP level prior to TACE and LT, donor age, BMI, graft type, tumor number in explants, intra-operative blood loss, intraoperative infusion volume≥8,000ml, operative time, histological grade and microvascular invasion. Univariate analyses identified the following prognostic factors that predicted poor overall survival: recipient BMI≥28, AFP ≥400ng/ml prior to TACE, donor BMI≥26, LDLT, TACE non-responders and the presence of microvascular invasion. Multivariate Cox regression analyses were performed for these significant factors and revealed that TACE non-responsiveness and the presence of microvascular invasion were significant risk factors for overall survival in HCC patients following LT.

**Table 4:** Univariate analyses showing contributors to overall survival and tumor-free survival following LT

Variables	N	Overall survival rate	Tumor-free survival rate
		P value	P value
Recipient age ≥60 (yes/no)	13/102	0.716	0.316
Recipient gender (M/F)	103/12	0.495	0.579
Recipient BMI≥28 (yes/no)	21/94	0.036^*^	0.080
Cause of liver diseases (HBV/HCV/no)	106/2/7	0.361	0.229
Child-Pugh Score (A/B/C)	41/58/16	0.656	0.599
ALB (≥35/28-35/≤28)	78/29/8	0.970	0.980
Creatinine ≥100μmol/L (yes/no)	10/105	0.674	0.543
NLR≥4 (yes/no)	46/69	0.577	0.646
AFP ≥400ng/ml prior to TACE (yes/no)	51/64	0.008^*^	0.114
AFP ≥400ng/ml prior to LT (yes/no)	40/75	0.068	0.148
Donor age ≥40(yes/no)	25/90	0.232	0.417
Donor BMI≥26 (yes/no)	4/111	0.004^*^	0.014^*^
Graft type (LDLT/DDLT)	12/103	0.001^*^	0.001^*^
Tumor number in explant(1/2-3/multiple)	61/43/11	0.514	0.940
Tumor diameter≥5cm (yes/no)	72/43	0.524	0.074
Milan criteria satisfied (yes/no)	58/57	0.397	0.627
UCSF criteria satisfied (yes/no)	74/41	0.393	0.379
TACE responders (responder/non-responder)	64/51	0.024^*^	0.019^*^
TACE times (1/2-3/multiple)	30/74/11	0.311	0.076
Intra-operative blood loss≥1,500ml (yes/no)	55/60	0.519	0.578
Intraoperative infusion volume≥8,000ml (yes/no)	73/42	0.263	0.133
Operative time≥10 h (yes/no)	32/83	0.618	0.420
Histological grade (well/moderate/poor/unknown)	45/39/21/10	0.734	0.593
Microvascular invasion (yes/no)	75/40	<0.001^*^	<0.001^*^

M: male; F: female; BMI: body mass index; HBV: hepatitis B virus; HCV: hepatitis C virus; MELD: model for end-stage liver disease;

NLR: neutrophil-lymphocyte ratio; AFP: alpha-fetoprotein; DDLT: deceased-donor liver transplantation; LDLT: living-donor liver transplantation; UCSF: University of California, San Francisco; TACE: transarterial chemoembolization

**Table 5:** Multivariate analyses of factors contributing to overall survival and tumor-free survival rates

Variables	Hazard ratio	95% CI	P value
Prognostic factors of overall survival			
Recipient BMI≥28	1.328	0.876-1.542	0.321
AFP ≥400ng/ml prior to TACE	1.661	0.971-2.326	0.102
Donor BMI≥26	1.472	0.848-1.643	0.557
LDLT	1.328	0.897-1.762	0.210
TACE non-responder	4.018	1.689-8.452	<0.001^*^
With microvascular invasion	2.218	1.908-3.563	<0.001^*^
Prognostic factors of tumor-free survival			
Donor BMI≥26	1.221	0.891-1.452	0.115
LDLT	1.287	0.916-1.676	0.357
TACE non-responder	5.110	2.782-10.284	<0.001^*^
With microvascular invasion	2.466	1.721-4.872	<0.001^*^

BMI: body mass index; AFP: alpha-fetoprotein; LDLT: living-donor liver transplantation; TACE: transarterial chemoembolization

As shown in Tables 4 and 5, univariate analyses were also performed to analyze factors predictive of tumor-free survival rates. Donor BMI≥26, LDLT, TACE non-responsiveness and the presence of microvascular invasion were identified as significant factors contributing to the tumor-free survival rate. Multivariate analysis of the ten factors found to be significant in the univariate analysis confirmed TACE non-responsiveness and the presence of microvascular invasion as significant contributors to tumor-free survival.

## DISCUSSION

LT is considered the best therapeutic option for select HCC patients. The currently accepted worldwide selection criteria for HCC LT are the Milan criteria. Even with a considerable 5-year survival rate that is higher than 70%, the Milan criteria have been considered too strict in that they restrict the inclusion of HCC patients who are outside the LT list but would benefit from this procedure. Many groups all over the world have attempted to expand these criteria with respect to tumor diameter or number; as a result, the UCSF criteria [6], Pittsburgh criteria [16], Navarra criteria [17], Valencia criteria [18] and others [19] have been proposed. Furthermore, many groups have suggested that total tumor volume should be proposed as a useful parameter to describe tumor burden in HCC patients awaiting liver transplantation [8, 20]. Recently, Zheng SS proposed the Hangzhou criteria, which include tumor diameter, histological level and AFP level. The Hangzhou criteria were the first to combine tumor diameter with the biological characteristics of the tumor. Nevertheless, the Hangzhou criteria have shortcomings, as these criteria remain inclusion criteria, and the histological level may only be obtained by biopsy, which may result in needle-track seeding or bleeding [21, 22]. Thus, the Milan criteria remain the most accurate inclusion criteria for HCC LT patients. For patients whose HCC are outside the Milan criteria, the inclusion criteria for HCC LT include additional risk factors, such as tumor number, diameter, volume, histological level and micro-RNA level [6, 7, 9], although no criterion includes all of these risk factors. In addition, grading of post-operative complications requires many definitions and comparisons. However, in 2004, Clavien et et al.[23] proposed the Clavien system to evaluate the grade of surgical complications, which provided a stream-lined classification system for post-operative complications, and the Clavien or Claven-do system is currently used all over the world. Pre-LT locoregional therapies, such as resection, radiofrequency ablation (RFA), TACE and TACI, among others, may successfully evaluate tumor characteristics. In particular, TACE, due to its safety and efficacy, should be considered in selection criteria as a potentially effective tool for prioritizing HCC patients for LT [15]. Based on the Barcelona Clinic Liver Cancer (BCLC) guidelines for the treatment of HCC, TACE was introduced as a first-line non-curative treatment for intermediate (BCLC-B) HCCs [24]. However, in clinical practice, some patients with early stage (BCLC-0 or A) HCC and contraindications for surgery or local treatments or cases with a shortage of liver grafts for LT have been treated with TACE. TACE has been proven safe and effective as a down-staging or bridging therapy for HCC patients awaiting LT [25, 26]; however, in this current study, our main goal was not to demonstrate the potential of TACE as a down-staging or bridging modality but to identify possible preoperative parameters that could be used as biological selection criteria for HCC LT. In our retrospective study, we analyzed the long-term clinical outcomes in 115 patients who fulfilled the Hangzhou criteria and underwent TACE followed by LT.

Our current analysis should be considered in the context of the variable results of several reports showing that responses to TACE served as a predictor of outcome following LT. However, there are some potentially important improvements in our study when compared to these previous reports [3, 8, 10, 11, 13]. First, we graded the toxicity of TACE based on World Health Organization criteria; the majority of toxicities were grade 1 or 2, with only a few patients suffering grade 3 toxicity, and no grade 4 toxicities were observed. Thus, Pre-LT TACE was a relatively safe procedure when compared to RFA or resection [27]. Second, we assessed the response to TACE using mRECIST rather than RECIST criteria [15, 28]. Third, when we compared the responders and non-responders, we noted that multiple tumor targets, an absence of arterial-phase enhancement in enhanced imaging scans, NLR ≥4 and microvascular invasion were associated with TACE failures prior to LT. Furthermore, we combined the TACE responses and the Milan criteria and noted that in the Milan criteria subgroups, the responders and non-responders showed no significant differences in long-term outcomes. However, for HCCs outside of the Milan criteria, the responders showed much better long-term outcomes than non-responders, indicating that the response to TACE may be more effectively applied to HCCs outside the Milan criteria. Lastly, in the subgroup analysis, the majority of responders were identified after the first or second TACE treatments, whereas three or more TACE treatments did not achieve a response in most HCC patients. Moreover, the long-term outcomes of first- or second-session responders were much better than those of responders who received three or more sessions of TACE.

Terzi [29] first compared the long-term outcomes of responders to first-session and repeated-session TACE responders and noted that the rates of CR and recurrence following the first and second TACE sessions were similar and that the majority of patients achieved a response by the second TACE treatment, with only a few patients submitted for three or more TACE sessions. These findings are consistent with those of the current study, as our data analyses indicated 15 of 115 cases (13.0%) achieved HCC CR after the first two TACE sessions; however, only 2 of 34 cases (5.9%) achieved this outcome after receiving three or more TACE sessions. In addition, 57 of 115 patients with HCC were classified as responders (50.0%) after the first or second TACE session, whereas 7 of 34 patients (20.6%) achieved this outcome after receiving 3 or more TACE sessions. These data are consistent with the literature in studies in which the World Health Organization criteria were used, in which CR rates were reported at 2-6%[30]. However, in Terzi’s report [29], approximately 50% of patients submitted to TACE treatment achieved CR after the first session, and in the randomized trial of Lammer et al.[31], the authors reported a 20% CR rate in patients receiving 3 courses of TACE. The reasons for these differences may be the different criteria used for evaluating tumor responses, the selectivity of the technique and the expertise of the radiological center; however, the main reason for these differences should be the tumor characteristics, which lead to a large differences in the inclusion criteria for TACE. The latency between TACE and evaluation may also affect the rate of CR or PR; in some patients, this period was one year, whereas in our study, all re-TACE patients were submitted ‘on demand’ (meaning: necessary). The fact that more TACE sessions were required in the non-responder group compared to the responder group, as shown in Table 2 also supports these points.

In our univariate and multivariate analyses, all clinical and laboratory data were considered, and the Milan criteria, UCSF criteria, AFP level, NLR, recipient and donor characteristics, and intra-operative data were not significant predictors of survival or recurrence in our limited series of patients. In contrast, TACE response and microvascular invasion were the most predictive factors for HCC patient overall or tumor-free survival. However, the predictivity of microvascular invasion cannot be routinely utilized for selecting patients for LT because the presence or absence of vascular invasion is not typically known prior to LT. In contrast, the response to TACE may be assessed preoperatively. Indeed, the efficacy of TACE as a predictive factor was more marked in HCCs extending outside the Milan criteria; in patients who achieved CR or PR, we observed more than 75% 5-year survival following LT, whereas the survival and recurrence-free survival rates were dramatically lower in non-responders. The advantages of this predictor were more obvious when HCCs were outside the Milan criteria, and the responder group showed significantly higher overall and tumor-free survival rates than the non-responders. However, survival rates were comparable when HCCs were within the Milan criteria, which is in contrast to the results of Kim’s study [11], in which 5-year HCC recurrence was much lower in patients responding to TACE than in patients who did not respond (HCC patients were within the Milan criteria). Our results may be explained by the fact that the efficacy in down-staging (responder) HCCs may be greater for larger tumors [32] and that the error when evaluating changes in tumor diameter or number may be more obvious for larger tumors. Although Bargellini [13] demonstrated the effectiveness of the TACE response when based on mRECIST guidelines, all patients in his study had HCCs that were outside the Milan criteria, and no HCCs within the Milan criteria were included; thus, it is likely that many of these patients would benefit from LT. In our study, we included all patients accepted for TACE prior to LT, and our analysis was more comprehensive and far-reaching when compared to other reports. Although the response to TACE in predicting long-term outcome involved patients outside the Milan criteria, the responders (CR or PR) whose HCCs were outside the Milan criteria showed similar survival rates as patients who were within the Milan criteria, as shown in Figure 3, which supports Yao’s report [6].

A previous study by Georgiades et al.[33] demonstrated that 50% of patients with HCC who did not respond to initial TACE ultimately responded and showed improved survival following a second course. Sieghart et al.[34] also proposed that the Assessment for Retreatment with TACE(ART) score, which depends upon tumor response and impairment in liver function, should be used to identify patients who are unlikely to benefit from a second TACE session. However, in our cases, the long-term outcomes of responders to the first or second TACE treatment were much better than those of patients who achieved a response after receiving 3 or more TACE sessions; in particular, 19 HCCs were classified as CR or PR (i.e., responders) after the first TACE session, 38 patients after the second TACE session, 4 after the third TACE session and 3 after 4-6 TACE sessions (as shown in Figure 1). The overall survival rate and tumor-free survival rate were compared between the first- or second-session TACE responder group and responders receiving 3 or more TACE sessions, and the former group showed much better 1-, 3- and 5-year overall survival rates than the latter group (P=0.024; shown in Figure 4A). In addition, the 1-, 3- and 5-year tumor-free survival rates were also better in the first-or second-session TACE responder group compared with responders receiving 3 or more TACE sessions, although the P value was 0.080 (as shown in Figure 4B). Due to the poorer outcomes observed in patients receiving more than two sessions, TACE should only be used once or twice. These data are in contrast to other reports that suggested repeating TACE for HCC patients when TACE was used as a selection criterion for HCC [29]. The primary reason for this difference may be that previous studies only compared the outcomes of responders and non-responders who were submitted for more than two sessions of TACE, whereas our study compared responder outcomes in groups receiving no more than two sessions of TACE with those receiving more than two sessions of TACE. The following three points may further explain our results. First, an initial or second TACE session may be sufficient to select for biological characteristics, whereas repeated TACE sessions may not change the tumor characteristics. Second, multiple sessions of TACE may exert unfavorable effects on liver function, as shown in Table 1. Lastly, the efficacy of TACE depends primarily on the vascularity, and in particular, the arterial characteristics of the tumor; thus, whereas the first or second session of TACE may embolize the majority of arteries in the tumor, repeated sessions are unnecessary except for when new nutrient arteries occur, which are indicative of progressive disease [35]. Thus, when used as a biological selection criterion, practitioners should proceed very cautiously when using three or more sessions of TACE. Our results support those of Jung [36], showing that the clinical outcomes of initial responders were notably better than those of patients who required more procedures to achieve a response and were improved compared to already established persistent non-responders. Lai Q, et al [37] has performed a multiple center analysis in European, and the results is consistent with ours’, meanwhile, we are performing a analysis with multiple centers from China mainland with larger patient number. Meanwhile, A recent meta analysis have reviewed 15 studies [38], and demonstrated statistically significant differences in survival and recurrence between different RECIST criteria after LT.

There are several limitations to our study. First, the primary limitation of our study relates to its retrospective setting and the fact that it involved only a limited series of patients who underwent TACE and LT. Second, the study included only patients who underwent TACE prior to LT. Pre-LT liver resection, RFA and other pre-LT therapies, which may also act as selective methods for LT, were not included in our current study [15]. Third, an interval between TACE and LT of at least 6 months is required to identify disease progression after an initial adequate tumor response to TACE, and in our series, the mean interval was shorter than 6 months. This short interval was primarily due to timely availability of liver grafts while HCC patients remained within the Hangzhou criteria, which are the inclusion criteria used in China.

In summary, based on the results of our analyses, we suggest the following combination of inclusion criteria for HCCs: when within the Milan criteria, HCC patients may be submitted for LT where the overall survival rate is almost 70% at 5years; when outside the Milan criteria, HCC patients may also be submitted for LT if the HCC responds (CR or PR) to pre-LT TACE, particularly after the first two sessions. However, a prospective, large cohort, multiple center study is required to validate the role and relevance of this parameter.

## MATERIALS AND METHODS

### Study populations

We retrospectively reviewed the medical and radiology records of all HCC patients who underwent transplantation in our center from Dec 2002 to Dec 2014. The preoperative diagnosis of HCC was based on AASLD guidelines [39]. Non-invasive criteria for a diagnosis of HCC in cirrhotic liver cases included the presence of a nodule that was larger than 1 cm with contrast medium uptake during the arterial phase and washout during the portal-venous or late-venous phase. If these characteristics were not obvious, dynamic contrast-enhanced ultrasonography or magnetic resonance imaging (MRI) was recommended to confirm the diagnosis of HCC. Any transplanted patients revealing incidental cancer upon histological examination of the explanted liver were excluded from this analysis, and the details of the inclusion and exclusion criteria are shown in Table 6. Patients were immediately listed if they fulfilled the Hangzhou criteria based on imaging but accepted TACE as a bridging therapy; those patients whose HCC did not satisfy the Hangzhou criteria were recommended for TACE as a down-staging therapy. TACE was repeated if there was incomplete necrosis, tumor re-growth, or the appearance of new lesions. Written, informed consent was obtained prior to TACE and LT. This retrospective study was approved by the local ethics committee. Institutional review board approval was not required for this retrospective analysis.

**Table 6:** Primary inclusion/exclusion criteria for this study

Inclusion criteria
Primary hepatocellular carcinoma
Age from 18 to 70 years
Liver cirrhosis
Accepting pre-LT TACE
BCLC-0,A or B HCCs
**Exclusion criteria**
BCLC-C HCCs (with the presence of macrovascular invasion or extrahepatic metastasis)
Accepting any other adjuvant therapy solely or combined, such as resection or RFA, among others
With no or insufficient clinical data for evaluatingthe response after TACE
Follow-up lost
The interval from TACE to LT was too short to evaluate the effectiveness of TACE (less than 1 month)

### TACE protocol

All TACE procedures in our center were performed by one of three interventional radiologists with at least 10 years of experience in interventional radiology. Depending on the tumor size, location and arterial supply of the tumor, a 3 Fr microcatheter (Microferret; Cool, Bloomington, IN, USA) was advanced toward the tumor-feeding arteries for selective embolization using transfemoral access, and TACE of the feeding arteries was performed by further super-selective catheterization as close to the tumor as possible. A mixture of doxorubicin hydrochloride (Adriamycin; Ildong Co. Ltd., Seoul, Korea) and an emulsion of iodized oil (Lipiodol; Laboratorie Guerbet, Aulnay Sous Bois, France) was injected until complete blockage of the tumor-feeding branch was demonstrated. The dose of embolization agent was determined based on tumor size, tumor number, feeding vessels and liver function status. Following embolization, angiography was performed to determine the extent of vascular occlusion and to assess blood flow in other arterial vessels.

### Image evaluation and analysis following TACE

At one month after each adjuvant therapy, an enhanced CT or MRI scan was performed to evaluate the tumor radiological response. A follow-up enhanced imaging scan was performed every 1-2 months during the first half year and every 3-5 months thereafter in all cases to assess recurrence. Enhanced computed tomography (CT) or MRI images were evaluated by radiologists and reviewed by liver transplant surgeons with more than 10 years of experience in the application and interpretation of pre-LT adjuvant therapies based on the mRECIST guidelines as follows. If there was irregular nodular enhancement or central necrosis of the lesion, the largest dimension of contiguous enhancement was measured. For patients who underwent more than one adjuvant pre-LT therapy, their response to TACE was determined by comparing the relative arterial-phase tumor enhancement diameters on the most recent and the initial contrast-enhanced images. The decrease or increase in the sum of the diameters of arterially enhanced target lesions was defined as the sum changes of all targets. Full lipiodol uptake area should not be considered as necrosis.

The following mRECIST response categories are based on the relative arterial-phase tumor enhancement and/or sum of unidimensional diameters in enhanced CT or MRI scans [11, 40].

(1). Complete response (CR): disappearance of any intra-tumoral arterial enhancement in all target lesions.

(2). Partial response (PR): at least a 30% decrease in the sum of diameters of viable (contrast enhancement during arterial phase) target lesions.

(3). Stable disease (SD): tumor reaction not meeting CR/PR criteria but less than a 20% increase in arterial-enhancing lesions.

(4). Progressive disease (PD): an increase of at least 20% in the sum of the diameters of arterial-enhancing lesions or the formation of new nodules.

CR and PR cases were defined as responders, and SD and PD cases were defined as non-responders. Based on these grouping criteria, we divided our 115 patients into two groups: the responder group (64 cases) and the non-responder group (51 cases). The HCC patient demographics at baseline, their tumor characteristics and intraoperative and donor data were compared between the two groups, and we also compared long-term outcomes between the two groups, including overall survival and tumor-free survival.

### Liver transplantation

During follow-up, once a liver graft became available and provided the HCC remained within the Hangzhou criteria (without the presence of macrovascular invasion and fulfilling one of the two following items: (a) total tumor diameter of less than or equal to 8 cm or (b) total tumor diameter greater than 8 cm, histopathologic grade I or II and a preoperative AFP level of less than or equal to 400 ng/mL), the patient was submitted for the LT procedure. Chest radiography and bone scans were necessary prior to transplantation. LT was performed using classic orthotopic LT for deceased liver donors and the piggy-back technique for living donors (LDLT). Each organ donation or transplantation in our center strictly followed the guidelines of the Ethical Committee of West China Hospital, the regulations of the Organ Transplant Committee of Sichuan Province and the Declaration of Helsinki. No prisoner served as a donor in our center. For LDLT, the donation was voluntary and altruistic, and we informed the donors and their families of the possible risks of a donor hepatectomy. Written consent was provided by the donors for their information to be stored in the hospital database and used for research. Prior to transplantation, lamivudine therapy (100mg/day, orally) was initiated when hepatitis B virus was diagnosed using the serum hepatitis B virus deoxyribonucleic acid (HBV DNA) test. Post-LT managements has been introduced in our previous study [41].

### Follow-up assessments

The overall survival rate and tumor-free survival rate were the primary criteria examined during follow-up assessments. The efficacy of LT was evaluated at 1 month by contrast-enhanced CT or MRI and according to the levels of tumor markers (AFP), followed by every 2 to 3 months over the next year and every 5-6 months thereafter, to assess treatment outcomes. Chest radiography and bone scintigraphy were performed when extrahepatic HCC recurrences were suspected. Time to recurrence was defined as the interval between LT and the first confirmed recurrence. The overall follow-up time was defined as the interval between LT and either local tumor progression or the final follow-up. Patients were followed until death, 5 years after LT or the end date of this study.

### Statistical analysis

All data were analyzed using the SPSS statistical software package (SPSS Inc., Chicago, USA; version 17.0).Patient baseline characteristics and other continuous variables are expressed as the mean ±SD and were compared and calculated using non-parametric tests. Categorical data were formatted as frequencies and compared using the X^2^ test. HCC response to pre-LT adjuvant therapy was compared using Student’s t-test and the Chi-squared test. The overall survival and tumor-free survival rate were defined as the time interval between LT and death or tumor recurrence and were estimated using the Kaplan-Meier method and compared using the log-rank test for significance. Univariate analyses were performed to identify factors predicting overall and tumor-free survival. All variables with P<0.05 were included in the multivariate analysis to assess independent predictor factors using Cox regressions. All tests were considered significant if P<0.05.

## Abbreviations

HCC: Hepatocellular carcinoma
LT: liver transplantation
CR: complete response
PR: partial response
SD: stable disease
PD: progressive disease
BCLC: Barcelona Clinic Liver Cancer
TACE: transarterial chemoembolization
NLR: Neutrophil-lymphocyte ratio
UNOS: United Network for Organ Sharing
EASL: European Association for the Study of the Liver
AASLD: American Association for the Study of Liver Diseases
MRI: magnetic resonance imaging
CT: computed tomography
HBV DNA: hepatitis B virus deoxyribonucleic acid
HBIG: hepatitis B immunoglobulin
BMI: body mass index
HBV: hepatitis B virus
HCV: hepatitis C virus
MELD: Model for end-stage liver disease
DDLT: deceased-donor liver transplantation
LDLT: living-donor liver transplantation
UCSF: University of California, San Francisco
AFP: alpha-fetoprotein
ON: overall necrosis
BMI: body mass index
HBV: hepatitis B virus
HCV: hepatitis C virus
MELD: model for end-stage liver disease
RFA: radiofrequency ablation
